# Evidence of the Role of Inflammation and the Hormonal Environment in the Pathogenesis of Adrenal Myelolipomas in Congenital Adrenal Hyperplasia

**DOI:** 10.3390/ijms25052543

**Published:** 2024-02-22

**Authors:** Vipula Kolli, Emily Frucci, Isabela Werneck da Cunha, James R. Iben, Sun A. Kim, Ashwini Mallappa, Tianwei Li, Fabio Rueda Faucz, Electron Kebebew, Naris Nilubol, Martha M. Quezado, Deborah P. Merke

**Affiliations:** 1 National Institutes of Health Clinical Center, 10 Center Drive, Bethesda, MD 20892, USAdmerke@cc.nih.gov (D.P.M.); 2 Laboratory of Pathology, National Cancer Institute, Bethesda, MD 20892, USAsunapathol@gmail.com (S.A.K.);; 3 D’Or Institute for Research and Education (IDOR), São Paulo 05403, Brazil; 4 The Eunice Kennedy Shriver National Institute of Child Health and Human Development, Bethesda, MD 20892, USAfabio.faucz@nih.gov (F.R.F.); 5 Division of General Surgery, Department of Surgery, Stanford University School of Medicine, Palo Alto, CA 94304, USA; 6 National Cancer Institute, Bethesda, MD 20892, USA

**Keywords:** congenital adrenal hyperplasia, adrenal myelolipoma, cell migration, inflammation, RNA-sequencing, B-lymphocytes

## Abstract

Adrenal myelolipomas (AML) are composed of mature adipose and hematopoietic components. They represent approximately 3 percent of adrenal tumors and are commonly found in patients with congenital adrenal hyperplasia (CAH). CAH provides a unique environment to explore AML pathogenesis. We aimed to evaluate the role of the immune system and hormones that accumulate in poorly controlled CAH in the development of AML. When compared to normal adrenal tissue, CAH-affected adrenal tissue and myelolipomas showed an increased expression of inflammatory cells (*CD68, IL2Rbeta*), stem cells (*CD117*) B cells (*IRF4*), and adipogenic markers (*aP2/FABP4, AdipoQ, PPARγ, Leptin, CideA*), and immunostaining showed nodular lymphocytic accumulation. Immunohistochemistry staining revealed a higher density of inflammatory cells (CD20, CD3, CD68) in CAH compared to non-CAH myelolipomas. In vitro RNA-sequencing studies using NCI-H295R adrenocortical cells with exogenous exposure to ACTH, testosterone, and 17-hydroxyprogesterone hormones, showed the differential expression of genes involved in cell cycle progression, phosphorylation, and tumorigenesis. Migration of B-lymphocytes was initiated after the hormonal treatment of adrenocortical cells using the Boyden chamber chemotaxis assay, indicating a possible hormonal influence on triggering inflammation and the development of myelolipomas. These findings demonstrate the important role of inflammation and the hormonal milieu in the development of AML in CAH.

## 1. Introduction

Adrenal myelolipomas are rare, benign lipomatous tumors of the adrenal gland, composed of a varying mixture of mature adipose tissue and hematopoietic components of myeloid and erythroid cells [1,2]. Although rare, adrenal myelolipomas are one of the most common tumors of the adrenal and represent an important consideration in the evaluation and management of adrenal tumors. Other lipomatous adrenal tumors include lipomas, teratomas, angiomyolipoma, liposarcoma, hibernoma, and rare adrenocortical tumors with a fat component [3]. Adrenal myelolipoma occurs equally in both sexes and increased incidence with age has been observed [2,4]. Previous case reports suggest that a typical myelolipoma contains lipid tissue with an active bone marrow component that is associated with normal hematopoiesis, but with an increased megakaryocyte number [5,6]. Although the etiology of myelolipoma development is not fully understood, a well-known and accepted theory is that they originate from the reticulo-endothelial stem cell rests of blood capillaries in the adrenal [6,7].

The common occurrence of myelolipomas (mostly unilateral) in congenital adrenal hyperplasia (CAH) patients is well documented [8]. CAH due to 21-hydroxylase deficiency is an autosomal recessive disorder of adrenal steroidogenesis characterized by cortisol and aldosterone deficiencies and ACTH-driven adrenal androgen excess. In general, patients with CAH are predisposed to adrenal tumor formation, especially in untreated or undertreated patients [8,9]. Approximately 25–35 percent of adrenal tumors in patients with CAH are myelolipomas [10]. The high incidence of myelolipomas in patients with poorly controlled CAH suggests that ACTH has a role in stimulating tumor formation. A large size can cause a mass effect requiring surgical removal [2,11], and traumatic spontaneous retroperitoneal hemorrhage has been described [12]. Some adrenal myelolipomas have also been described in patients with cancer and diabetes mellitus, supporting the possible role of the hypothalamic–pituitary–adrenal stress response [3].

In the present study, we investigated the role of the immune system and the CAH hormonal milieu in myelolipoma formation. We characterized the inflammatory and lipogenic aspects of the CAH adrenal and myelolipoma in vivo through immunohistochemistry and gene expression analysis, and in vitro using an established adrenocortical cell line. The hormonal profile of patients with CAH and adrenal myelolipoma typically demonstrates high levels of ACTH, C21, and C19 hormones, and our study is the first to investigate the effects of these high levels of hormones on inflammatory cells in adrenocortical cells.

## 2. Results

### 2.1. Characteristics of the CAH Study Population

Adrenal and myelolipoma tissue was obtained from 4 (3 female, 1 male; age range 43 to 69 years) patients with CAH caused by a 21-hydroxylase deficiency. All had the classic, simple virilizing phenotype with a diagnosis beyond infancy. Three patients had adrenalectomy performed due to chronic abdominal pain, with the myelolipoma’s largest dimension ranging from 7 to 13.5 cm. Adrenal and myelolipoma tissue was obtained from one patient during an autopsy. All patients had a history of noncompliance with glucocorticoid and mineralocorticoid replacement therapy for several years.

### 2.2. Histological Features of CAH Adrenal Myelolipoma

**Figure 1:**
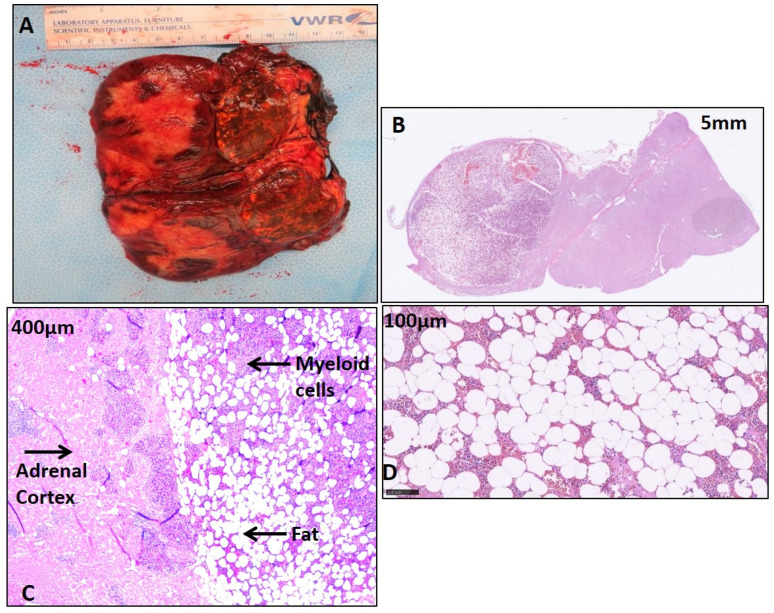
Representative gross and histopathology of a myelolipoma of one of the CAH patients. (**A**) Gross image shows a mass replacing the normal adrenal and composed of soft yellow and brown tissues measuring about 11–12 cm. (**B**,**C**) H&E (hematoxylin and eosin)-stained sections show the myelolipoma and adjacent hyperplastic adrenal gland (**C**) H&E-stained sections show disorganized hyperplastic adrenal cortical cells with islands of myeloid cells, (**D**) H&E-stained sections show the mature adipose tissue.

**Figure 2:**
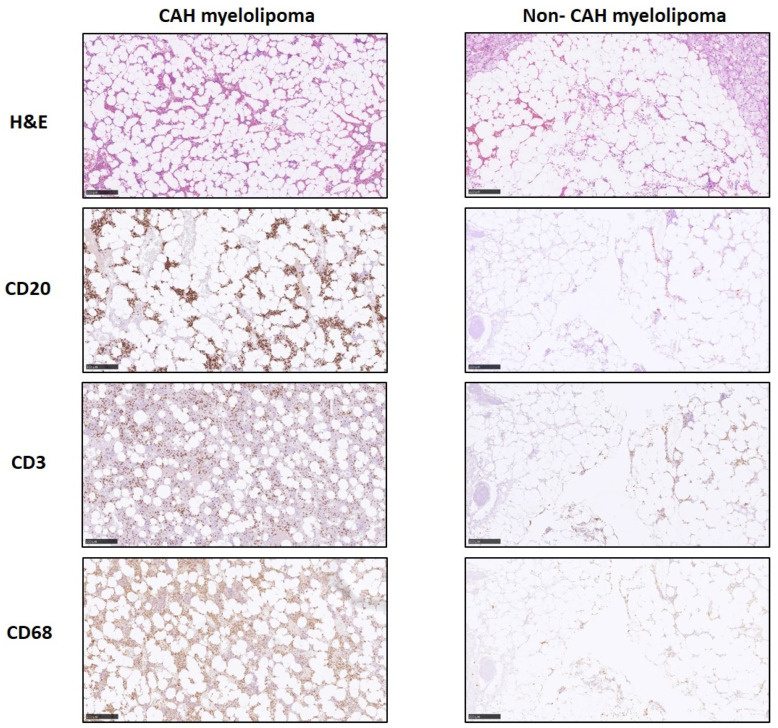
H&E and immunohistochemistry of myelolipoma from CAH and non-CAH patients (original magnification X100). CAH myelolipoma shows a prominent lymphocytic infiltration forming aggregates/clusters. (B-lymphocytes (CD20), T-lymphocytes (CD3), and Monocytes/Macrophage lineage (CD68). Non-CAH myelolipoma shows less lymphocytic infiltrates.

The CAH adrenal glands had a disorganized hyperplastic adrenal cortical region (Figure 1A). As expected, histopathology of the CAH adrenal myelolipoma showed a characteristic adipose component and abundant hematopoietic tissue comprising all three bone marrow elements [13], including megakaryocytes [5] (Figure 1B,C). In addition, the myelolipoma tissue contained islets of cells comprising a mature and precursor myeloid lineage (Figure 1C,D). Nodular lymphocytic infiltration was present only in the CAH adrenal myelolipoma (Figure 2) with positive staining of cell surface inflammatory markers including CD20 and CD3 specific for B and T cell surface proteins, respectively, and CD68 specific for macrophages/monocytes. Overall, a higher density of inflammation was observed in the CAH adrenal myelolipoma compared to non-CAH adrenal myelolipoma.

### 2.3. Gene Expression of Inflammatory and Lipogenic Genes by qRT-PCR

**Figure 3:**
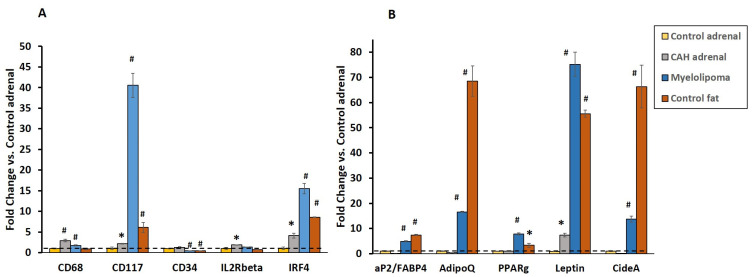
(**A**). Elevated expression of inflammatory genes in CAH adrenal and myelolipoma tissue indicates the presence of inflammation. Real-time PCR analysis of the indicated genes in the control adrenal, CAH adrenal, myelolipoma and control fat; mean ± SEM; vs. control adrenal. All genes were normalized to 18S RNA. *p*-value: * *p* ≤ 0.05, ^#^ *p* ≤ 0.001. (**B**). Elevated expression of adipocytic genes in myelolipoma tissue shows the presence of mature lipoid tissue in CAH adrenal. Real-time PCR analysis of the indicated genes in the control adrenal, CAH adrenal, myelolipoma and control fat; mean ± SEM; vs control adrenal. All genes were normalized to 18S RNA. *p*-value: * *p* ≤ 0.05, ^#^ *p* ≤ 0.001.

Cell surface inflammatory markers were expressed in both the CAH adrenal and myelolipoma tissue. The *CD68* and *CD117* transcripts were significantly higher (*p* < 0.005) in the CAH adrenal and myelolipoma compared to the normal adrenal (Figure 3A). However, the transcripts of *IL2Rβ*, which play an important role in the T cell-mediated immune response, were detected in CAH adrenals, but not in the myelolipoma. Expression of *IRF4*, a lymphocyte-specific interferon regulatory transcription factor involved in tumorigenesis, was also upregulated in CAH adrenals and myelolipoma compared to normal tissues (*p* < 0.001) (Figure 3A). The expression of *CD34*, a stem cell marker, was downregulated in the myelolipoma and normal fat compared to adrenal tissues, indicating the presence of mature fat or terminally differentiated adipocytes in the myelolipoma (Figure 3A).

As expected, the expression of the adipocytic genes aP2/fatty acid binding protein (*FABP4*), adiponectin and adipokine (*AdipoQ*), transcription factor *PPARγ,* and leptin in myelolipoma tissue revealed the presence of mature lipoid tissue in the myelolipoma compared to both CAH and control adrenal tissues. Similarly, expression of *Cide-A*, a cell death activator protein A, which is a lipid droplet-specific binding protein, was upregulated in myelolipoma and not in both CAH and control adrenals (*p* < 0.001) (Figure 3B).

### 2.4. RNA-Sequencing Analysis

**Figure 4:**
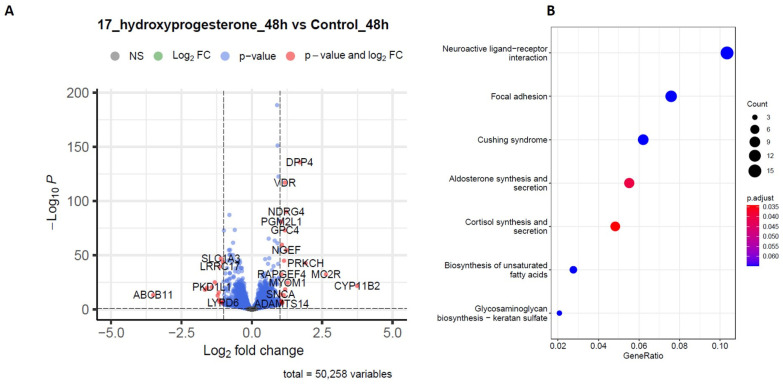
Comparative transcriptome profiles of NCI-H295R cells treated with 17 OHP for 48 h. (**A**) Differential expression genes (DEG) analysis: volcano plot of differentially expressed genes between cells treated for 48 h with 17OHP and the control. (**B**) KEGG pathway enrichment analysis dot plot (produced by ClusterProfiler).

Studies were performed to assess the steroid hormone-induced effects on NCI-H295R adrenocortical cells. Transcriptomic analysis on the cells after exogenous exposure to 17-hydroxyprogesterone (17OHP) (10 µM) for 48 h was performed and compared to the control treatment. The RNA-Seq differential expression of genes (DEGs) data showed a high abundance of gene transcripts involved in cell cycle progression, and proliferation (*PRKCH, NGEF, PGM2L1, NDRG4*) in cells treated with 17OHP when compared to the control treatment (Figure 4A). Gene ontology and KEGG analyses were used to explore the mechanisms and potential biological pathways involved in exogenous steroid treatment. GO enrichment analysis showed that DEGs are mainly involved in biological functions including cell junction assembly, cell-matrix adhesion, the negative regulation of T cell apoptosis, thermogenesis regulation, the stress response, axon development, and membrane potential regulation. To further study the possible pathways directly affected by the exogenous steroidal treatment of adrenocortical cells, target genes were classified by KEGG pathway enrichment analysis and functional enrichment using the R (v 4.2.1) software ClusterProfiler (v3.15). The terms with a *p*-value less than 0.01 were determined to be significantly enriched. The top 7 related signaling pathways which were below the default significant threshold and the corresponding 57 enriched targets were obtained. These targets involved a variety of signaling pathways including steroidogenesis, fatty acid biosynthesis, and focal adhesion (Figure 4B).

### 2.5. Cell Chemotactic Assay

**Figure 5:**
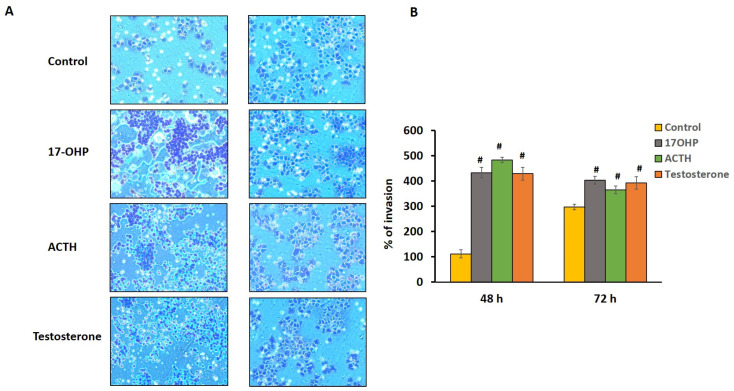
NCI-H295R cell invasion of B-lymphocytes in the presence of elevated hormone levels. (**A**) After 48 h and 72 h of hormonal treatment, migrated cells were stained and counted (original magnification X50). (**B**) bar graphs showing the quantitative analyses of the invasion of cells after hormonal treatment, respectively. Data represented as mean ± SD, ^#^ *p* ≤ 0.001.

B cell invasion or migration capability in response to the adrenocortical cells induced with different hormone (17OHP, testosterone, and ACTH) treatments was measured at 48 h and 72 h using BioCoat Matrigel invasion chambers. Exposure to 17OHP, ACTH, and testosterone for 48 h and 72 h significantly increased B cell migration (Figure 5A). The percentage of B cell migration/invasion increased after 48 h and 72 h treatments with 17OHP (48 h, *p* ≤ 0.001; 72 h, *p* ≤ 0.001), ACTH (48 h, *p* ≤ 0.001; 72 h, *p* ≤ 0.001), and testosterone (48 h, *p* ≤ 0.001; 72 h, *p* ≤ 0.001) (Figure 5B).

## 3. Discussion

In our current study, we demonstrate nodular lymphocytic infiltration in the myelolipomas of CAH patients. To our knowledge, this is the first study to analyze the expression of inflammatory and adipocytic genes in the adrenocortical myelolipomas of CAH patients. We show in vitro that adrenocortical cells exposed to the hormonal milieu typical of an untreated CAH patient display an increase in *PRKCH, PGM2L1, LYPD6, NDRG4*, and *ADAMTS14*, factors involved in tumorigenesis, cell cycle progression, and regulation. Moreover, we show that adrenocortical cells treated with high levels of hormones that accumulate in poorly controlled CAH initiate the migration of B-lymphocytes.

Adrenal myelolipomas are rare, benign lipomatous tumors of the adrenal gland, but constitute approximately 3–4% of primary adrenal tumors, 6–16% of adrenal incidentalomas, and are the second most common cause of adrenal neoplasms after adrenal adenomas [2,3]. Although adrenal myelolipomas are commonly found in patients with poorly controlled congenital adrenal hyperplasia (CAH), the pathogenesis of adrenal myelolipoma is unknown. Our data support the theory introduced in 1950 that adrenal tissue has common precursor progenitor cells for both hematopoietic and mesenchymal lineages, especially adipose and immune cells [14]. We previously reported a lack of zonation [15] and lymphocytic infiltration [16] in the adrenals of patients with classic CAH.

Steroid secretion in the normal human adrenal gland is regulated by a complex network of autocrine/paracrine interactions between adrenocortical cells, endothelial cells, nerve terminals, and chromaffin cells. Cells of the immune system, including lymphocytes, macrophages and mast cells, have been described in the normal adrenal cortex [17]. Our data also indicated a nodular infiltration of B-lymphocytes along with increased T-lymphocytes in CAH myelolipomas. We found a greater gene expression of the inflammatory cytokine markers *CD68* (monocytes/macrophages) and *CD117* (mast cells/stem cell) in the CAH adrenal and myelolipoma compared to the normal adrenal, suggesting that inflammation may play a role in the poorly controlled CAH adrenal and possibly in the formation of myelolipoma. Our results show a lessor density of inflammatory cells in non-CAH adrenal myelolipoma compared to CAH adrenal myelolipoma tissues, which might be due to a lack of intra-adrenal cortisol secretion in CAH-affected adrenals. Autocrine and paracrine interactions within the adrenal gland have been shown to regulate adrenal function during both physiological and pathophysiological conditions [18,19,20]. The adrenal cortex contains an extensive vascular network, which supports a paracrine interaction between different cell types [18,21,22]. We speculate that the hormonal milieu of the poorly controlled CAH adrenal stimulates progenitor cells to differentiate into adipocytes, resulting in fat accumulation, with inflammation driven by fat-laden M2-macrophages.

Human adrenals secrete large amounts of inactive steroid precursors which are converted into potent androgens and estrogens in target peripheral tissues [23]. This steroid conversion is a multienzyme-step machine that is hindered in adrenal disorders of steroid metabolism, and new data suggests a role of these precursors in the etiology of adrenal tumors and also as potential biomarkers [24]. Emerging data reveals that the synthesis of the precursor molecule dehydroepiandrosterone (DHEA) occurs in an ordered manner, and the sulfated form of DHEA (DHEAS), a major product of adrenal zona reticularis, is the most abundantly found steroid hormone in blood along with other steroid sulfates, where steroid sulfation represents an alternate approach to regulate the action of the steroidal hormones [24]. The sulfation pathways, representing the dynamic process of regulating the steroid hormones, have also been suggested to play an important role in steroid hormone-dependent cancers. A strong role of these sulfates in the pathophysiology of adrenal tumors has been suggested, and these sulfates and steroid precursors may have clinical utility as diagnostic biomarkers [24]. However, DHEA is not elevated in CAH [25]. Deficient 21-hydroxylase activity shunts cortisol precursors to other steroids, leading to the production of excess adrenal androgens [11]. New biomarkers, such as 11-oxygenated androgens, are being studied in the diagnosis and management of CAH [26], but biomarkers of adrenal tumor formation have yet to be investigated.

Several studies have described the molecular mechanisms that lead to the formation of adrenocortical tumors [27]. Overall, there are two types of adrenocortical tumors: functional (hormone-secreting) and silent, and these are categorized as either malignant or benign. Benign hormone secreting adenomas can cause Cushing’s syndrome, primary aldosteronism or less commonly virilization [28,29]. The majority of adrenocortical carcinomas are active and produce hormones, especially in children, and adrenal hormone precursors are often useful diagnostic biomarkers [28,29,30,31]. Myelolipomas are benign and nonfunctional but can be found along with functional tumors [32].

Although adrenocortical hormones and their precursors are often elevated in the presence of adrenocortical tumors, the impact of circulating steroids on tumor formation is mostly unknown. ACTH, including intra-adrenal ACTH, has been shown to be a major player in the pathogenesis of adrenocortical neoplasms [33]. Interestingly, adrenal myelolipomas have not been reported in Addison’s disease, where there is autoimmune destruction of the adrenals and ACTH is very high [32]. To investigate the etiology of adrenal myelolipoma, we designed an in vitro model using adrenocortical cells and mimicked the critical features of the tumor microenvironment. In poorly controlled CAH patients, there is a chronic stimulation of the adrenal cortex by ACTH, leading to hyperplasia, and an excess production of adrenocortical androgens and steroid precursors. Our study is the first to investigate the effects of these high levels of hormones on an in vitro cell model. We found significant migration of immune cells in response to a CAH-like hormonal environment, supporting the hypothesis that the hormonal milieu plays a role in initiating active inflammation in the adrenals leading to the development of myelolipoma.

Multiple studies have shown that sex steroids, including estrogens, progesterone and androgens, influence immune cell response [34]. The innate immune system, comprising many different subtypes of myeloid progenitor cells, expresses the androgen receptor, and testosterone has been shown to have a direct effect on early myelopoiesis [35]. Many epidemiological studies have found sex differences in immunity [36]; men are more susceptible to infections (bacteria and viruses) than females, possibly due to the elevated levels of pro-inflammatory cytokines and their responses [37]. In elderly men, one other problem that is prevailing is impaired wound healing, possibly due to the pro-inflammatory actions of testosterone on histiocytes [36]. In contrast, in women, there is approximately a twofold higher prevalence of autoimmune disorders compared to men, possibly due to the role of testosterone-mediated neuroprotection [38,39]. Though androgens predominantly have an anti-inflammatory effect by inhibiting immune cell reactions, elevated testosterone in women has been associated with an increased risk of a subset of ovarian cancers [40] and an increased risk of breast cancer [41,42]. However, elevated levels of androgens are commonly found in women with classic CAH, and increased instances of cancer has not been observed.

Progesterone, in addition to 17OHP, is elevated in CAH. Both bind to human mineralocorticoid receptors [43,44], and both activate the glucocorticoid receptor [45]. 17OHP has weak progestational activity, and progesterone has been shown to have immunomodulatory effects [46]. Progesterone receptors are not expressed in all immune cells, but progesterone mediates the maternal immune responses during pregnancy through glucocorticoid receptors in T cells [46,47]. In men, progesterone has also been shown to influence the immune system [48].

Our RNA sequencing results suggest that exogenous high steroidal treatment of adrenocortical cells, especially with 17OHP, activates genes involved in cell proliferation and progression. ADAMTS14 protein, a metallopeptidase that degrades the extracellular matrix and its components, and thus increases cell migration and invasion, was found to be expressed in the tumor cells [49,50]. Increased expression of *ADAMTS14* in our study signifies the role of steroids in cell invasion. One other gene found to be highly expressed in cells treated with hormones was *DPP4.* Recent studies indicate that DPP4 has an important role in immune regulation, and has been suggested to be a prognostic marker for malignancy [51,52]. Our transcriptional data also revealed an increase in the expression of genes involved in cell cycle progression, proliferation, phosphorylation, and tumorigenesis, suggesting that hormones may be playing a role in the progression of the adrenal lesion. Although benign, adrenal myelolipomas are often quite large, with an average size of 10.2 cm at the time of diagnosis [53].

ACTH likely plays a role in myelolipoma formation; however, we found a decreased expression of the *ACTH receptor* (*MC2R*) and androgen receptor (AR) in myelolipoma tissue, and an increased expression in CAH adrenal tissue. This is in contrast to a prior report [54]. We meticulously and manually teased out myelolipoma from the adrenal cortex which was confirmed by H&E and various antibody staining techniques. In addition, the expression of *Cide-A*, a cell death activator protein A, was significantly (*p* < 0.001) upregulated in myelolipoma, but not in the adrenal, confirming that there was no cross-contamination of the adrenal cortex and myelolipoma tissues. Furthermore, we observed low *PTEN* expression in both CAH adrenal and myelolipoma tissues, indicating that the decreased expression of the tumor suppressor *PTEN* gene is associated with the loss of its tumor suppressor functions, thus leading to uncontrolled cell growth causing tumor development. Although murine adipocyte cell lines express *MC2R*, and ACTH can regulate adipocyte function [33], human mature adipocytes only express low levels of *MC2R*, and ACTH does not regulate lipolysis in mature human adipose tissue [55], supporting our findings that inflammatory cells and adrenocortical hormones play a role in myelolipoma formation in CAH.

The limitations of our study include the potential bias of our population. Patients with CAH who underwent adrenal surgery had a history of poor disease control, with prolonged exposure to high levels of ACTH and adrenocortical steroids. The inclusion of one autopsy AML is also a limitation, as rapid autopsy tissue quality is not comparable to fresh surgical tissue collection. Moreover, we evaluated a limited number of adrenocortical steroids, and did not study hormones individually. Further studies are needed to evaluate the effect of additional adrenocortical steroids on immune cell migration, and to potentially translate in vitro findings into clinical practice. Additionally, the cell line used for our in vitro studies is an adrenal cancer cell line, and thus may not be the best model to accurately reflect the adrenocortical response to hormone treatment. However, the H295R cell line is the most commonly and widely used cellular model for in vitro experiments, as this unique cell line retains the ability to express genes that encode for all of the important enzymes involved in steroidogenesis.

In summary, we identified unique characteristics of the CAH adrenal myelolipoma, demonstrating that active inflammation likely plays a role in the propensity towards adrenal tumor formation. We found increased inflammation in the CAH adrenal myelolipoma compared to the non-CAH adrenal myelolipoma, the expression of inflammatory and adipocytic genes in the adrenocortical myelolipoma of CAH patients, and in vitro adrenocortical cells exposed to the hormonal milieu typical of an undertreated CAH patient displayed an increase in the factors involved in tumorigenesis, cell cycle progression, and regulation. We found that in addition to ACTH, excess androgens and androgen precursors have the ability to stimulate adrenocortical cells and recruit lymphocytes. This finding has important clinical implications in the development of therapies for adrenal tumors, especially those found in patients with CAH, and also in the identification of reliable biomarkers which can be crucial for diagnosis, prognosis, and monitoring the progression of tumor formation in CAH.

## 4. Materials and Methods

### 4.1. Tissue Collection

Tissue samples were collected from four CAH patients enrolled in a National History Study at the National Institutes of Health (NIH) Clinical Center, Bethesda, MD, USA (NCT#00250159) and from non-CAH patients (3 Cushing’s, 1 pheochromocytoma) enrolled in a National Cancer Institute (NCI, Bethesda, MD, USA) protocol (Protocol # NCI-09C0242). Studies were approved by the NIH Institutional Review Board. Written informed consent was obtained. Along with four non-CAH adrenal myelolipomas which were used as controls, normal adrenal and adipose tissue from a de-identified tissue bank (Laboratory of Pathology, NCI, Bethesda, MD, USA) were also used as controls. All tissues were reviewed by one pathologist (MMQ). Immediately after surgery, tumor samples were fixed in formalin, dehydrated and embedded in paraffin. Part of the sample was frozen in liquid nitrogen and stored at −80 °C until total RNA was isolated.

### 4.2. Histology and Immunohistochemistry

Multiple tissue sections with a thickness 5 µm from formalin-fixed paraffin-embedded (FFPE) tissue blocks of CAH adrenal, normal adrenal, normal adipose tissue, and tumor myelolipoma tissue sections were mounted on Superfrost plus slides (Erie Scientific, Ramsey, MN, USA) and hematoxylin and eosin (H&E) staining was performed on tissue sections from CAH adrenal, normal adrenal, and adrenal myelolipoma, as previously described [16]. Histopathological assessment of inflammation and expression of inflammatory markers was performed using prediluted antibodies as markers (Roche Tissue Diagnostics, Marlborough, MA, USA), CD3 (T lymphocyte-specific) (790–4341: clone 2GV6), CD20 (B-lymphocyte-specific) (760–2531: clone L26), and CD68 (macrophage/monocyte histiocyte-specific (790–2931: clone KP-1). Appropriate negative and positive controls were used. H&E and all other IHC slides were viewed at ×40, ×100, ×200, and ×400 magnification.

### 4.3. Gene Expression Analysis by qRT-PCR

Total RNA was extracted from the unstained microdissected tissue slides for each sample using the Qiagen RNeasy FFPE kit (QIAGEN, Valencia, CA, USA) according to the manufacturer’s instructions. After determining the RNA yield and quality using Nanodrop 2000/2000c spectrophotometers (Thermo Fisher Scientific, Waltham, MA, USA), complementary DNA (cDNA) was generated from 0.75 ug of RNA using the Transcriptor First Strand cDNA Synthesis Kit (Roche, Indianapolis, IN, USA) according to the manufacturer’s instructions. The comparative C_T_ method was used to calculate the relative expression of various genes. 18*S* RNA and *GAPDH* expression values were used to normalize each sample. Then, qRT-PCR was performed using the Power SYBR Green PCR Master mix (Applied Biosystems, Carlsbad, CA, USA) and processed using the ABI 7300 system (Applied Biosystems, Carlsbad, CA, USA). Relative gene expression was measured using the ∆∆−Ct method. Primers were designed using Primer3 v 4.1.0 software, Boston, MA, USA. Normal fat was used to normalize the adipocytic genes. Primer sequences are available upon request.

### 4.4. Cell Culture

NCI-H295R (ATCC CRL-2128, ATCC, Manassas, VA, USA), human adrenal corticocarcinoma cells, were maintained in DMEM: F12 medium (ATCC, Manassas, VA, USA) supplemented with 2% fetal bovine serum (Invitrogen, Carlsbad, CA, USA), in the presence of 1% insulin, transferrin, selenium, and linoleic acid in the form of ITS + Premix (Corning, NY, USA), 2.5% Nu-Serum (Corning, NY, USA) and 1% penicillin/streptomycin solution. For cell migration assays, Raji (CCL-86) B-lymphoblast cells were maintained in complete media with RPMI-1640, 10% FBS and 1% penicillin/streptomycin solution. All the cells were incubated at 37 °C with 5% CO_2_.

### 4.5. RNA-Sequencing and Differential Expression Testing

H295R cells were maintained in DMEM: F12 complete media. Cells were divided and seeded in six-well cell culture plates in triplicates and then treated with hormones (17OHP: 10 μM, ACTH: 0.68 nM, testosterone: 100 nM). Cells were treated for 72 h and total RNA was isolated, quality and quantity were checked using a Bioanalyzer, sequencing libraries were constructed via the TruSeq stranded RNA kit, and RNA-sequencing was performed as described previously [16]. The output data files were used to identify and compare any differentially expressed genes using DESeq2 (v3.16). The Gene Ontology (GO) of biological functions and Kyoto Encyclopedia of Genes and Genomes (KEGG) pathway enrichment analyses were performed using ClusterProfiler (v3.15) [56] on the subset of differentially expressed genes. Values were considered significant when the *p*-value was < 0.05. Enrichment dot plots were created for KEGG pathways for the *Homo sapiens* using ClusterProfiler and the KEGG database.

### 4.6. In Vitro Invasion and Transfilter Migration Assays

The Corning BioCoat Matrigel Invasion Chamber system (Cat no. 354480, Corning, NY, USA) was used to measure cell migration or invasion. NCI-H295R cells were seeded in 24-well plates. After 24 h of incubation at 37 °C with 5% CO_2_, cells were treated with hormones (17OHP: 10 μM, ACTH: 0.68 nM, testosterone: 100 nM) and BioCoat Matrigel inserts containing Raji cells (B-lymphocytes) were added on to the wells. After incubation for 48 h and 72 h at 37 °C, inserts were removed from the culture, fixed, and stained with a Diff- Quik kit (Cat no. B4132-1A, Allegiance Chemicals LLC, Cockeysville, MD, USA). The number of cells that migrated through the Matrigel-coated membrane was counted. Invasion of the B-lymphocytes influenced by hormonally induced adrenocortical cells was calculated as the percentage of the cells that migrated through the Matrigel insert membrane relative to the percentage of cells that migrated through the membrane in the control (no hormone exposure).

### 4.7. Statistical Analysis

All ex vivo testing and in vitro experiments were performed in triplicates. Statistical differences between the cell treatment conditions were analyzed using the paired Student’s *t* test. All data represent the means and standard deviation of the means (SD). *p*-values are two-tailed and the statistical significance was set to * *p* ≤ 0.05. ^#^ *p* ≤ 0.001.

## Data Availability

The data sets generated during the current study are not publicly available but are available from the corresponding author on reasonable request.
